# DEVELOPMENT OF AN INTERGENERATIONAL AND AGE-FRIENDLY COMMUNITY: APPLICATION OF THE SOCIAL CAPITAL FRAMEWORK

**DOI:** 10.1093/geroni/igad104.0008

**Published:** 2023-12-21

**Authors:** Youjung Lee, Jacqueline McGinley

**Affiliations:** Binghamton University - SUNY, Binghamton, New York, United States; Binghamton University - SUNY, Binghamton, New York, United States

## Abstract

In intergenerational programs, people from multiple generations share their talents, build healthy social relationships, and experience improved well-being and social presence and identity (Lee et al., 2021). As a part of age-friendly community initiative in a small city in the Northeast, an intergenerational engagement community project, named “Senior to Senior”, involved a multi-year partnership among community agencies, including the local Office for Aging, a university, a high school, a senior center, and a church. As part of this partnership, a community dance was held in May of 2022, where local high school students and older adults participated in a variety of intergenerational activities. This study explored how the principles of social capital are incorporated into the development of intergenerational and age-friendly communities. Using focus groups with community partners (n=6) and surveys with older adult participants (n=29), the research team sought to describe how community agencies collaborate on the development of intergenerational and age-friendly communities. Carpiano’s framework of social capital (2008) guided the data analysis and the development of following themes: (a) importance of social capital identification; (b) development of social cohesion among multiple generations; (c) assessment of social antecedent for the development of intergenerational and age-friendly community; and, (d) outcomes of the community events on diverse stakeholders. The findings highlight the importance of understanding the contextual factors of each community and the agencies within, while also elucidating the necessity of stakeholder buy-in for the sustainable development of intergenerational and age-friendly communities.

